# A Solitary Malignant Schwannoma in the Choana and Nasal Septum

**DOI:** 10.1155/2014/202910

**Published:** 2014-09-09

**Authors:** Eun Jung Lee, Kee Jae Song, Yeon Suk Seo, Kyung-Su Kim

**Affiliations:** Department of Otorhinolaryngology, Gangnam Severance Hospital, Yonsei University College of Medicine, 211 Eonju-ro, Gangnam-gu, Seoul 135-720, Republic of Korea

## Abstract

Malignant schwannoma is an extremely rare tumor and the risk of malignant schwannoma increases in patients with von Recklinghausen's disease. Recently, we encountered a case of solitary malignant schwannoma in the choana and posterior nasal septum. Malignant schwannoma has not been previously reported in these locations. A 53-year-old man, who was immunologically healthy and showed no abnormal dermatological lesions, presented with a polypoid mass in the right nasal cavity and underwent endoscopic mass excision. The mass originated from the choana and the posterior portion of the right nasal septum. This mass was confirmed as a malignant schwannoma on histological examination and immunohistochemical staining. After endoscopic excision, postoperative adjuvant radiotherapy was administered, and there was no recurrence at 1 year after treatment. This case suggests that a solitary malignant schwannoma should be considered in the differential diagnosis of a mass in the posterior nasal cavity.

## 1. Introduction

Malignant schwannomas represent approximately 10% of soft tissue sarcomas and are rarely found in the head and neck region. The most common locations are the extremities, trunk, chest, and retroperitoneum [1, 2]. Malignant schwannomas in the paranasal sinuses or nasal cavity are exceedingly rare and there have only been 21 cases of malignant schwannoma in the nasal cavity and paranasal sinuses reported worldwide [3–9]. One case of malignant schwannoma in the nasal septum has been reported, and the origin of the tumor was the anterior and middle portion of the septum [10]. However, a malignant schwannoma originating from the choana and posterior nasal septum has not been reported. Multiple forms of malignant schwannomas are common and occur in association with von Recklinghausen's disease, but solitary forms are extremely rare [4]. Recently, we experienced a case of solitary malignant schwannoma in the choana and the posterior nasal septum; this schwannoma was not associated with von Recklinghausen's disease.

## 2. Case Report

**Figure 1:**
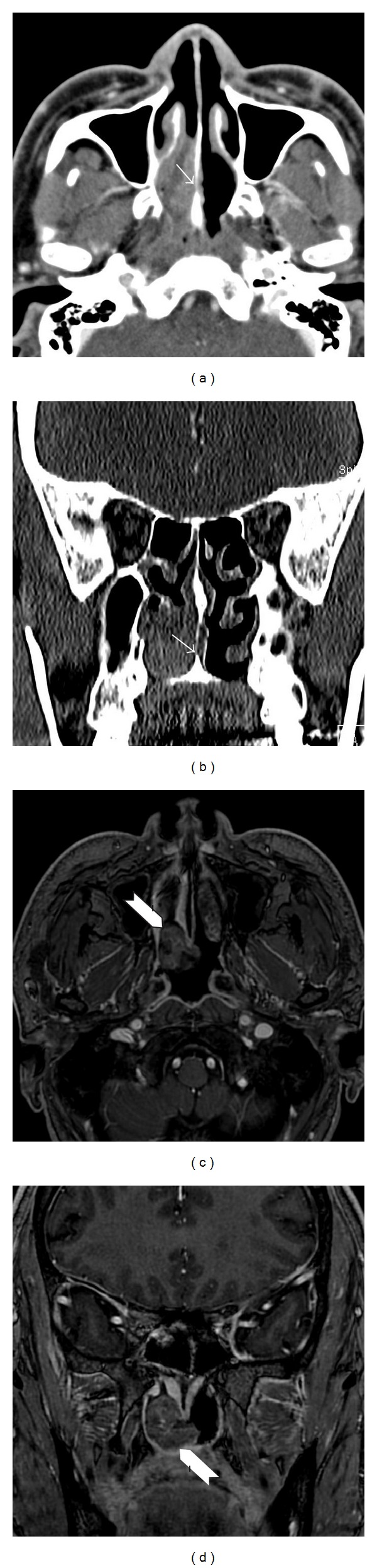
Axial (a) and coronal (b) images of paranasal sinus computed tomography. The mass shows a heterogeneous density between the nasal septum and lateral nasal wall, with erosion of the septal bone (arrow). Axial (c) and coronal (d) images of paranasal sinus T1-gadolinium-enhanced magnetic resonance imaging. A 2.5 × 1.5 cm sized, gadolinium-enhanced fungating mass with internal hemorrhage, and vascularity (arrowhead) can be seen between the right choana and lateral wall. The lesion is well demarcated with smooth margins.

**Figure 2:**
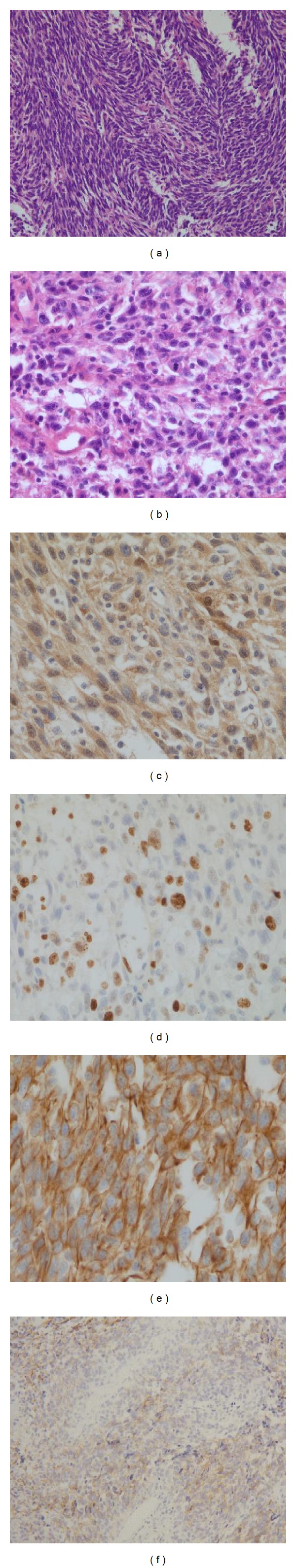
Histopathological findings of hematoxylin and eosin staining ((a), (b)) and immunohistochemical staining ((c)–(f)). (a) A vaguely whorled growth pattern with large and multilobulated nuclei, resembling a palisade (×200). (b) The individual neoplastic cells have finger-like projections of cytoplasm and atypical multilobulated nuclei with irregular nuclear membranes (×400). (c) Brown-colored cells are positive for S-100 protein (×400). (d) Ki-67-positive cells show brown-colored nuclear staining (×200). (e) Ubiquitous intermediate filaments of vimentin are positively expressed as brown color (×400). (f) Brown-colored cells are positive for CD56 (×100).

A 53-year-old man presented with nasal obstruction and frequent epistaxis that had persisted for 3 months. He had well-controlled hypertension and diabetes mellitus. No abnormal dermatological spots were found on the body and the serological test for human immunodeficiency virus was negative. Endoscopic examination revealed a polypoid mass extending from the midportion of the right inferior turbinate to the choana. Images of paranasal sinus computed tomography (PNS CT) revealed a 2.5 cm long and heterogeneously enhanced mass filling the right nasal cavity with extension to the right choana and nasopharynx and some erosion of the septal bone (Figures 1(a) and 1(b)). Paranasal sinus magnetic resonance imaging (PNS MRI) showed a well demarcated, 2.5 × 1.5 cm-sized, gadolinium-enhanced, and fungating mass with internal hemorrhage and increased vascularity (Figures 1(c) and 1(d)). The mass was endoscopically excised using a piecemeal technique and a soft, pinkish, and friable mass originating from the right choana and posteroinferior portion of the nasal septum was obtained. The adjacent septal mucosa was removed but septal bone was preserved. Histologically, the mass was composed of elongated spindle-shaped cells arranged in short bundles with large and multilobulated nuclei, resembling a palisade (Figure 2(a)). The individual neoplastic cells had finger-like projections of cytoplasm and atypical nuclei with irregular nuclear membranes (Figure 2(b)). The diagnosis was confirmed by positive immunohistochemical staining for S-100 protein in nodular proliferating spindle cells (Figure 2(c)). Ki-67 labeling index was seen in ~30% of tumor cells, indicating a high degree of mitosis (Figure 2(d)). Also, the tumor cells were positive for vimentin and CD56 (Figures 2(e) and 2(f)). However, the tumor cells were negative for creatine kinase (CK), leukocyte common antigen (LCA), CD 34 (endothelial cell marker), smooth muscle antigen (SMA), chromogranin, and synaptophysin. Thus, the histopathological diagnosis was malignant schwannoma. A postoperative positron emission tomography (PET)-CT scan showed no abnormal fluorodeoxyglucose uptake in the nasal cavities. Adjuvant radiotherapy of 59.4 Gy was administered to the tumor bed area for 2 months at 1.8 Gy per fraction. The patient has been disease-free with no evidence of recurrence after 1-year follow-up.

## 3. Discussion

In the present case, the patient did not have acquired immune deficiency syndrome or any dermatologic abnormalities such as café-au-lait spots that are suggestive of von Recklinghausen's disease. Therefore, this case is the first reported case of a solitary malignant schwannoma confined to the choana and posterior portion of the nasal septum. The diagnosis of malignant schwannoma is confirmed by hematoxylin and eosin staining as well as immunohistochemical staining with specific markers that are expressed in Schwann cells. Histologically, malignant schwannoma has a high cellularity with spindle-shaped cells arranged in bundles, palisades, or whirls. Spindle-shaped cells arranged in sweeping fascicles have a moderate amount of cytoplasm and large multilobulated nuclei. In addition, dense cellular areas alternate with hypocellular and myxoid zones [11, 12]. Schwannomas usually exhibit intense immunostaining for S-100 protein (particularly in Antoni A areas), which is traditionally regarded as the best marker for malignant schwannoma. However, it has limited diagnostic utility as being positive in only about 50–90% of the tumor. Malignant schwannomas show positive staining for vimentin and CD56 and negative staining for CK, LCA, CD34, SMA, desmin, chromogranin, and synaptophysin. CD56 is expressed in about 50% of the tumors and it is used to distinguish from other neuroendocrine tumors and vimentin is used to distinguish from other sarcomas [11, 12]. In the present case, findings of hematoxylin and eosin staining and immunohistochemistry were compatible with those of malignant schwannoma. Many diseases are included in the differential diagnosis of malignant schwannoma in the nasal cavity, including sarcomatoid carcinoma, melanoma, atypical fibroxanthoma, malignant fibrous histiocytoma, rhabdomyosarcoma, and leiomyosarcoma [2].

Imaging features of malignant schwannoma are generally nonspecific. Benign schwannomas are usually well encapsulated, whereas malignant schwannomas may show extensive infiltration. Bone destruction can occur secondary to pressure erosion; therefore, such a finding does not necessarily indicate malignancy [1]. Intralesional hemorrhage has been reported in cases of adenocarcinoma with fungal infections [13]. In the present case, a fungating mass with internal hemorrhage and increased vascularity was noted on PNS MRI, but these findings were not specific for malignant schwannoma.

Treatment modalities for malignant schwannoma consist of surgical excision, systemic chemotherapy, or radiotherapy. Complete surgical excision with a safety margin is the mainstay treatment in patients with localized diseases. The role of radiotherapy and chemotherapy remains controversial; however, radiotherapy has been indicated in cases in which the tumor cannot be completely resected [5]. In the present case, we were unable to determine the presence of a malignant tumor using preoperative CT and MRI findings. Therefore, after the final pathological diagnosis, postoperative PET-CT was performed and postoperative adjuvant radiotherapy was initiated. Although the mass was excised using a piecemeal technique, it is likely that this patient may have a favorable prognosis because the tumor was solitary and confined to the choana and nasal septum and was not associated with von Recklinghausen's disease; furthermore, the postoperative PET-CT scan showed negative findings. However, if any signs of malignancy could be recognized preoperatively or intraoperatively, a more radical method of surgery such as septectomy should have been considered in this case. Presently, the patient has been disease-free with no evidence of recurrence 1 year after the treatment.

## 4. Conclusions

From our review of the literature, it is the first report of malignant schwannoma arising from septum extending to the posterior choana. Although the mass was excised using a piecemeal technique, it is likely that this patient may have a favorable prognosis because the tumor was solitary and confined to the choana and nasal septum and was not associated with von Recklinghausen's disease.
